# Virus-Induced MicroRNA Modulation and Systemic Sclerosis Disease

**DOI:** 10.3390/biomedicines12061360

**Published:** 2024-06-19

**Authors:** Irene Soffritti, Maria D’Accolti, Francesca Bini, Eleonora Mazziga, Dario Di Luca, Clara Maccari, Maria-Cristina Arcangeletti, Elisabetta Caselli

**Affiliations:** 1 Section of Microbiology, Department of Chemical, Pharmaceutical and Agricultural Sciences and LTTA, University of Ferrara, 44121 Ferrara, Italy; irene.soffritti@unife.it (I.S.); maria.daccolti@unife.it (M.D.); francesca.bini@unife.it (F.B.); eleonora.mazziga@unife.it (E.M.); 2 CIAS Research Center, University of Ferrara, 44122 Ferrara, Italy; 3 Department of Medical Sciences, University of Ferrara, 44121 Ferrara, Italy; ddl@unife.it; 4 Laboratory of Microbiology and Virology, Department of Medicine and Surgery, University of Parma, 43126 Parma, Italy; clara.maccari@unipr.it (C.M.); mariacristina.arcangeletti@unipr.it (M.-C.A.)

**Keywords:** microRNA, Systemic Sclerosis, biomarkers, beta-herpesviruses

## Abstract

MicroRNAs (miRNAs) are short noncoding RNA sequences that regulate gene expression at the post-transcriptional level. They are involved in the regulation of multiple pathways, related to both physiological and pathological conditions, including autoimmune diseases, such as Systemic Sclerosis (SSc). Specifically, SSc is recognized as a complex and multifactorial disease, characterized by vascular abnormalities, immune dysfunction, and progressive fibrosis, affecting skin and internal organs. Among predisposing environmental triggers, evidence supports the roles of oxidative stress, chemical agents, and viral infections, mostly related to those sustained by beta-herpesviruses such as HCMV and HHV-6. Dysregulated levels of miRNA expression have been found in SSc patients compared to healthy controls, at both the intra- and extracellular levels, providing a sort of miRNA signature of the SSc disease. Notably, HCMV/HHV-6 viral infections were shown to modulate the miRNA profile, often superposing that observed in SSc, potentially promoting pathological pathways associated with SSc development. This review summarizes the main data regarding miRNA alterations in SSc disease, highlighting their potential as prognostic or diagnostic markers for SSc disease, and the impact of the putative SSc etiological agents on miRNA modulation.

## 1. Introduction

MicroRNAs (miRNAs) are a class of noncoding RNAs (ncRNAs), 19–25 nucleotides (nts) in length, which can modulate gene expression at the post-transcriptional level and are involved in a broad range of molecular mechanisms, both in physiological and pathological conditions [1]. Their fortuitous discovery in a *Caenorhabditis elegans* model, more than thirty years ago, opened the way to the studies of antisense RNA–RNA interactions and their regulatory effects on gene expression [2,3].

The synthesis of miRNAs occurs in various steps, finely regulated across different subcellular compartments [4]. Briefly, canonical miRNA biogenesis starts with transcription, mainly of intragenic DNA sequences, by RNA polymerase II [5], leading to long primary miRNAs (pri-miRNAs). Pri-miRNAs are then cleaved by the Drosha-DGCR8 (DiGeorge Syndrome Critical Region 8) nuclear complex to release hairpin-shaped precursor miRNAs (pre-miRNAs, about 60–70 nts in length). These intermediates are exported by the Exportin5-RanGTP complex out of the nucleus, where a second RNase complex, consisting of the ribonuclease Dicer in association with its cofactor named “Transactivation Response element RNA-Binding Protein” (Dicer-TRBP), cleaves the pre-miRNAs into ~22 nt miRNA duplexes, generating mature miRNAs. MiRNAs are able to modulate gene expression through both *cis*- and *trans*-regulatory pathways [6]. During the *cis*-regulation, one of the two strands of the mature miRNA duplex is incorporated into the Argonaute (AGO) proteins (AGO1–4 in humans) to form a miRNA-induced silencing complex (miRISC), which interacts with the 3′ untranslated region (3′ UTR) of target mRNAs to induce mRNA degradation and repress gene expression at the post-transcriptional level [7]. In addition, silencing effects on gene expression have been observed in the case of miRNA interaction with 5′ UTR, coding or promoter sequences [8]. In *trans*-regulation, miRNAs can inhibit protein translation during the early steps of translation by interacting and/or competing with several factors: RNA-binding proteins or enzymes (e.g., decapping enzymes, deadenylase, 3′ and 5′ exonucleases, and endonucleases) involved in the 43S preinitiation complex formation or affecting post-initiation mechanisms, translation machinery, or post-translational modifiers [6,9].

Moreover, miRNAs biogenesis may also occur via different non-canonical pathways, mainly grouped into Drosha/DGCR8-independent and Dicer-independent pathways, reflecting the dynamism and the flexibility of miRNA machineries [8].

As to the peculiar nomenclature system of mature miRNA, consisting of the suffixes -5p or -3p, it depends on the directionality (5′ end or 3′ end) of the precursor strand included in the miRISC complex; in addition, the miR or miR* annotations indicate the highly expressed “guide strand” (miR) or the less expressed “passenger strand” (miR*), respectively [10].

It has been observed that a single miRNA could have more than 200 target mRNAs, and the expression of a single gene can be regulated by multiple miRNAs [11,12,13].

Since their discovery, a growing body of evidence has supported their key role as epigenetic regulators of a variety of physiological processes, including cell development, proliferation, differentiation, and stress adaptation [14,15,16]. Interestingly, dysregulated miRNAs expression has been observed in pathological conditions, including several types of cancer, metabolic disorders, and cardiovascular and chronic autoimmune diseases (ADs) [6,15].

The latter represent a group of complex and chronic inflammatory pathologies mainly defined by an impairment in autoimmune tolerance and improper immune responses towards healthy tissues [17]. Multiple factors may be associated with the predisposition, development, and/or progression of ADs, including host genetic alteration, persistent immune activation/reactivation, and environmental stimuli, both chemical and biological [18]. Recently, published epidemiological data pointed out that ADs affect around one out of ten individuals (2–10% of the population), showing a significantly higher prevalence in women compared to men (13% and 7%, respectively) [19]. The most relevant ADs include Rheumatoid Arthritis (RA), Systemic Lupus Erythematosus (SLE), Multiple Sclerosis (MS), Type 1 Diabetes, and Systemic Sclerosis (SSc) [20]. The diagnosis and treatment of ADs, especially of rare diseases such as SSc, are often hampered by challenges due to the complexity of the identification of initial symptoms; the poor knowledge of the disease; and the lack of rapid, accurate, and universally recognized diagnostic markers. Therefore, in recent years, growing interest has been given to the use of miRNAs as potential biomarkers or innovative therapeutic approaches [6,21,22,23,24].

This review aims to provide an updated overview of miRNA alterations in SSc, highlighting their possible role, and their potential as specific prognostic/diagnostic markers for the disease. Also, the relation between miRNA modulation and the putative SSc etiological agents is explored, particularly focusing on viral agents considered putative SSc triggers.

## 2. Systemic Sclerosis: An Overview

Systemic Sclerosis (SSc), also known as scleroderma (MIM: 181750; ORPHA: 90291), is a systemic connective autoimmune disease characterized by diffuse fibrosis and vascular abnormalities affecting the connective tissue of skin and internal organs (in particular, the lungs, heart, esophagus, low gastrointestinal tract, and kidneys) [25,26,27,28]. SSc develops as a result of multiple pathogenic mechanisms, primarily the abnormal deposition of the extracellular matrix (ECM) by altered fibroblasts, together with diffuse microangiopathy, immune dysregulation, tissue fibrosis, and apoptosis [26,29,30]. SSc patients are clinically classified based on the European League Against Rheumatism (EULAR) and American College of Rheumatology (ACR) criteria [31,32,33]. Common early clinical manifestations include Raynaud’s phenomenon, digital ulcers, hardening and tightening of the skin, and up to severe lung and heart sclerodermic lesions [28]. Despite the high variability of individual symptoms, the disease is typically categorized as “limited cutaneous” (lcSSc) and “diffuse cutaneous” (dcSSc) based on the extent of skin fibrosis: confined to finger/face/distal extremities with a slow progression in the case of lcSSc or affecting the trunk and proximal extremities with a rapid progression up to the fibrosis of internal organs in the case of dcSSc [26,34,35]. On these bases, the two subgroups present well-recognized patterns in disease severity and prognosis, with a higher risk for dcSSc patients to develop life-threatening complications, including pulmonary arterial hypertension, severe renal crisis, lung fibrosis, and heart and digestive tract involvement [36]. Other classifications also include SSc individuals without identifiable skin involvement (“SSc sine scleroderma”) or with a concurrent other fibrotic disease (“overlap syndrome”) [26,34,37]. A recent epidemiological study estimated the global incidence of the disease as 0.67 million (0.14–1.84) people annually and a prevalence of 18.87 per 100,000 persons (1.55–25.28) [38]. As for other ADs, SSc shows a marked prevalence in the female sex (F:M sex ratio around 4:1) in adults and in high-income level countries [36]. The etiology of SSc is still poorly understood, but several clinical, epidemiological, and experimental findings suggest that it is a multifactorial disease. Accordingly, research is heading towards elucidating several concomitant factors: genetic predisposition, oxidative stresses, and environmental triggers, including both toxic chemical agents (e.g., exposure to solvents and silica dust) and infectious agents [28,39].

So far, several mechanisms related to oxidative stress and redox regulation have been widely reported in SSc disease, related to inappropriate Reactive Oxygen Species (ROS) and Reactive Nitrogen Species (RNS) production, which can lead to pathological tissue damage [40].

In parallel, different infectious agents have been associated with SSc, including bacterial (e.g., *Helicobacter pylori* and *Mycoplasma*) and viral agents [28]. Among the viruses, those able to persist and reactivate in infected individuals were particularly claimed as possible triggers, including members of the *Orthoherpesviridae* family, such as Human Cytomegalovirus (HCMV), Human Herpesvirus-6A (HHV-6A) [28,41,42,43,44], and Epstein–Barr virus (EBV) [45,46,47], and members of the *Parvoviridae* family—in particular, Human parvovirus B19 (B19V) [48,49,50].

Overall, specific and periodic reactivation of viral infection, increased antibody and cell-mediated immune responses, and molecular mimicry represent the predominant mechanisms by which viral agents may be involved in the onset and progression of SSc [28].

**Figure 1:**
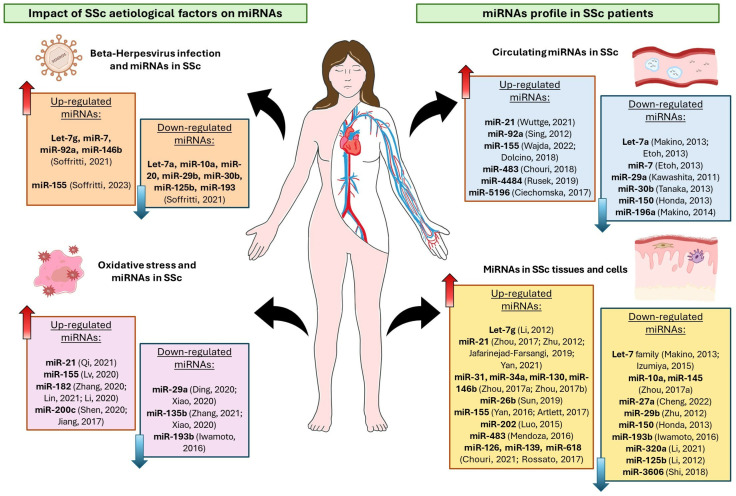
Profile of miRNAs deregulated in SSc disease, both at the tissue and extracellular level, and effects of oxidative stress and beta-herpesviruses infection on SSc-associated miRNAs [42,51,52,53,54,55,56,57,58,59,60,61,62,63,64,65,66,67,68,69,70,71,72,73,74,75,76,77,78,79,80,81,82,83,84,85,86,87,88,89,90,91,92] (SSc, Systemic Sclerosis; upward red arrows, up-regulated miRNAs; downward blue arrows, down-regulated miRNAs).

Interestingly, miRNAs could be induced and involved in oxidative stress and viral response processes; in these contexts, their deregulation can trigger or favor the development of multiple pathological pathways. Moreover, the epigenetic regulation driven by miRNAs has been recognized to have a significant role in controlling the main pathological pathways of SSc, including immune responses, autoimmunity, fibrosis, and vasculopathy. The present review aimed to define the miRNome profile characterizing SSc disease at both the tissue/intracellular and extracellular levels. In addition, the effects of two of the key etiological factors of SSc disease, oxidative stress and beta-herpesvirus infection, on miRNA expression and related impacts are presented in Figure 1.

## 3. MiRNAs and Systemic Sclerosis

MiRNAs can be classified into intracellular miRNAs and extracellular or circulating miRNAs [8]. Inside the cell, miRISC and target mRNA could localize in several subcellular compartments, including the nucleus, rough endoplasmic reticulum, mitochondria, Golgi apparatus, and cytoplasmatic vesicles, such as endosomes and lysosomes [8]. Moreover, extensive literature data have demonstrated miRNA release into the extracellular environment to mediate cell–cell communication, often associated with lipids and proteins, or secreted via exosomes [93,94,95]. Due to their stability in the extracellular environment, their presence has been evidenced in a broad range of biological fluids, including plasma, serum, saliva, tears, bronchial lavage, and breast milk [94,96].

Dysregulated miRNAs in SSc could have an inducing or repressing action, regulating the main pathways of the disease: microvascular changes, fibrosis, and immune alteration. Also, with regards to SSc, altered expression levels of miRNAs have been observed in both sclerodermic tissues and biofluids of SSc patients.

### 3.1. Alteration of MiRNA Profiles in SSc Tissues

Vascular impairment results are among the early hallmarks of SSc; this process leads to endothelial dysfunction and damage, worsened by tissue oxidative stress, hypoxia, and the recruitment of proinflammatory and profibrotic mediators. The key miRNA involved in SSc-related vasculopathy is miR-193b, which has been observed as decreased in SSc skin and fibroblasts [51]. This miRNA targets the uPA (urokinase-type plasminogen activator) gene, which deregulated expression induces angiogenesis impairment and enhances fibrotic and apoptotic pathways. Blood vessel injuries are typically marked by profound phenotypic and molecular alterations, involving, in particular, vascular smooth muscle cells (VSMCs), which undergo dedifferentiation and acquire a more proliferative and migratory phenotype [51].

The fibrotic process consists of the accumulation of a complex ECM composed of collagen, elastin, and fibronectin. The transforming growth factor (TGF)-β is a key profibrotic mediator, stimulating fibroblast proliferation and accumulation and ECM production [97]. The let-7 family miRNAs are highly conserved across animal species and the first observed miRNAs in humans [98,99]. A miRNA array analysis evidenced a significant downregulation of let-7 family miRNAs, particularly let-7a, in the skin of systemic and localized sclerodermic (LoSc) patients compared to normal and keloid skin samples [52]. Let-7a downmodulation was confirmed both in vitro and in a mouse model of bleomycin-induced dermal sclerosis and resulted in the excessive abnormal expression of type I collagen, typical of a profibrotic effect [52]. Another study reported that five let-7 family members (let-7a, let-7d, let-7e, let-7f, and let-7g) were downregulated in skin biopsies of patients with pulmonary hypertension (PH), one of the most severe complications of SSc. In addition, the let-7b and 7d levels were inversely correlated with the PH severity, highlighting the potential role of these skin miRNAs as biomarkers for PH severity in SSc subjects [53]. In contrast, Li and colleagues reported increased levels of let-7g in skin biopsies of SSc patients, which correlated with SSc pathogenesis [54]. An integrative analysis of miRNA–mRNA expression profiles in the skin of SSc patients highlighted that 21 miRNAs were differentially expressed in SSc compared to healthy controls; altered miRNAs were mainly involved in the Toll-like receptor, TGF-β, and Wnt signaling pathways [55]. Validation experiments confirmed that miR-146b, miR-130b, miR-21, miR-31, and miR-34a were highly expressed in SSc skin tissues and fibroblasts and in ECs after in vitro stimulation with SSc sera. On the contrary, miR-145 and miR-10a showed decreased levels in both SSc skin biopsies and primary skin fibroblasts and were deregulated after stimulation with SSc sera [55]. MiR-27a-3p was decreased both in SSc lung and skin tissues, which promoted fibrotic gene expression by the SPP1/ERK1/2 axis [56]. MiR-150 is another downregulated miRNA in skin and dermal fibroblasts at the plasmatic level [57]. Downregulation of this miRNA is linked to integrin β3-mediated TGF-β activation and led to the overexpression of type I collagen [57]. Zhu and colleagues reported increased levels of miR-21 and decreased amounts of miR-145 and miR-29b in skin biopsies and primary fibroblasts of lSSc and dSSc patients [58]. MiR-21, miR-145, and miR-29b predictively interacted with SMAD7, SMAD3, and COL1A1 mRNAs, respectively, potentially regulating the TGF-β pathway [58,100]. In particular, miR-21 is induced by TGF-β and, in turn, regulates TGF-β-mediated fibrogenic activation of skin fibroblasts by targeting Smad7. MiR-21 expression was also observed to increase in a SSc-bleomycin-induced mouse model, in which antifibrotic treatment with bortezomib restored the miR-21 and Smad7 levels [59]. In addition, miR-21 overexpression also affected SSc lung and dermal fibroblasts [59,60] and regulated Toll-like receptor signaling, suggesting its potential role in immune activation [101]. Both miR-130 and miR-202-3p were upregulated in skin tissue and fibroblasts of SSc patients, showing profibrotic activity by regulating TGF-β via antifibrotic PPARγ inhibition and MMP1 expression, respectively [61,62]. On the contrary, miR-320a has been reported to have an antifibrotic role. Its decreased levels have been observed both in fibrotic SSc patients with interstitial lung disease (ILD) complications and in a bleomycin-induced mouse model [62]. Its decrease in human pulmonary fibroblasts determined the inhibition of type I collagen deposition through the direct regulation of TGFBR2 (transforming growth factor beta receptor 2) and IGF1R (insulin-like growth factor 1 receptor) [63]. Moreover, SSc dermal tissue and primary fibroblasts contained significantly decreased levels of miR-125b and miR-3606-3p [54,64,102]. MiR-125b downregulation induced apoptosis and hindered proliferation and α-SMA expression in dermal fibroblasts cells [102]. MiR-3606-3p targets TGFβ-receptor type 2 (TGFBR2) and has been proposed as an antifibrotic modulator since its overexpression leads to a decline in the Smad2/3 levels and a decrease of type I collagen [64]. MiR-483-5p has been observed typically increased in fibroblasts and endothelial cells, where the expression of fibrosis-related genes is sustained, including collagen-type IV alpha 1 and 2 (COL4A1 and COL4A2) [65,103]. In addition, increased levels of miR-483-5p in endothelial cells lead to the increased transcription of two known myofibroblast differentiation markers (α-SMA and SM22A), promoting the transition to a myofibroblast phenotype [65]. MiR-155 is the second-most relevant miRNA in rheumatic diseases [6]. It was upregulated in SSc and LoSc skin lesions and correlated with the extent of fibrotic tissue damage [66]. Its profibrotic activity lies in the enhancement of Wnt/β-catenin and Akt pathways by the direct targeting of casein kinase 1a (CK1α) and src homology 2-containing inositol phosphatase-1 (SHIP1) [66]. In addition, treatment with antagomir-155 in mice results in a significant decrease of the extent of bleomycin-induced skin fibrosis, highlighting its role as a potential therapeutic target of SSc disease [66]. MiR-155 is also involved in SSc pulmonary fibrosis, being the most expressed miRNA in SSc lung fibroblasts [67]. Several data report its role in regulating both innate and adaptative immune responses [104,105].

The dysregulation of immune responses in SSc disease can be evidenced by the increased presence of inflammatory cells and mediators at the tissue level, together with the enhancement of type I interferon signature and autoimmune responses. MiR-155 SSc-related overexpression is promoted by inflammasome activation and leads to collagen production [67]. MiR-26b-5p, found upregulated in SSc fibroblasts, predictively targets CXCL9 and CXCL13 [68]. Of note, the miR-26b-5p inhibitor hinders the expression of cell fibrosis markers (α-SMA, fibroblast activation protein (FAP), collagen-type I alpha 2 (Col1A2), and Col4A1) through the NF-kB and JAK-STAT pathways in in vitro TGF-β-activated skin fibroblasts. Thus, this miRNA has been suggested as a promising target for SSc therapy [68]. Several miRNAs, including miR-618, miR-126, and miR-139-5p, act by unbalancing the immune response of SSc patients, particularly of plasmacytoid dendritic cells (pDCs). The upregulation of these miRNAs in SSc pDCs induces IFNα production and promotes IFN-inducing genes, causing SSc-pDC imbalance [69,70]. In particular, miR-618 targets an important transcriptional factor for pDC development and activation (IFN regulatory factor 8, IRF-8), and its upregulation in SSc dendritic cells inhibits pDC differentiation and promotes their ability to release IFNα [70]. The MiR-126 and miR-139-5p levels were positively correlated with the increased expression of IFN-responsive genes, suggesting the role of these miRNAs in the interferon response and immune dysregulation in SSc pathogenesis, even at the early stage of the disease [69]. Furthermore, miR-139-5p suppressed the expression of the deubiquitinating enzyme USP24, which is a cofactor for TLR7/9 signaling [69,106].

### 3.2. Alteration of Circulating MiRNAs in SSc

MiRNAs can be secreted into the extracellular environment, often associated with RNA-binding proteins or inside exosomal vesicles [93,94]. The profile and abundance of specific extracellular miRNAs can reflect the physiological condition of the subject; therefore, they have been widely suggested as potential biomarkers for difficult-to-diagnose diseases. The first data regarding the presence of circulating miRNAs in SSc disease involved miR-29a, which was first quantified in the serum of SSc patients, although no difference between SSc and healthy controls was observed [71]. Interestingly, a significant decrease of this miRNA was evidenced in a group of patients diagnosed as “scleroderma spectrum disorder” (SSD), who do not fit the ACR criteria for SSc but are at risk to develop the disease based on the points system proposed by Ihn et al., which assigns a global score based on five predisposing factors (extent of skin sclerosis, pulmonary abnormalities, antinuclear antibodies, pattern of Raynaud’s phenomenon, and nailfold bleeding) [71]. MiR-29a targets both α1(I) collagen and α2(I) collagen transcripts, so the dysregulation of type I collagens may be triggered by downmodulated miR-29a levels at the SSD stage but maintained by other miRNAs in SSc patients [71].

Further studies have observed a distinctive decreased expression of several miRNAs in different types of biofluids of SSc subjects, proposing their use as useful markers of the disease. Antifibrotic Let-7a showed a significant downregulation in the serum of SSc and LoSc patients compared to healthy controls, showing an inverse correlation with disease severity [52,72]. Similarly, miR-7 and miR-196a were expressed less in LSc sera compared to healthy subjects [72,73]. Of note, data concerning miR-7 expression reported conflicting findings [107]. Specifically, its downmodulation has also been observed locally in LSc dermal fibroblasts, and its general decrease is thought to contribute to LSc pathogenesis through the overexpression of α2(I) collagen protein [72]. On the other side, the microarray analysis reported increased levels of miR-7 in SSc dermal fibroblasts, which determined an antifibrotic effect, maybe as a negative feedback loop, although not sufficient, against excessive fibrogenesis [108]. MiR-196a has been reported under-expressed in LSc skin tissue and serum in vivo, and it is associated with the overexpression of type I collagen [73]. A quantitative PCR screening of 95 miRNAs targeting SSc-related genes by in silico predictive analysis highlighted a remarkable downregulation of about 95% of the tested miRNAs, and in particular, miR-30b were the most heavily decreased in SSc sera compared to healthy specimens, with levels inversely correlated with modified Rodnan skin scores [74]. The downmodulation was also confirmed in bleomycin-treated sclerotic skin in mouse model and in fibrotic skin of SSc patients [74]. This miRNA was observed to regulate PDGFR-β (platelet derived growth factor receptor beta) expression, thus suggesting the role of miR-30b in the pathogenetic PDGFR-β-driven pathway of SSc [74]. MiR-150 was observed underrepresented in SSc sera, compared to healthy individuals, and lower miRNA levels correlated with disease severity [57]. As observed in vitro in dermal fibroblasts, miR-150 acts by regulating integrin b3 and type 1 collagen expression, so its depletion induces an upregulation of these genes implicated in SSc pathogenesis [57].

Conversely, elevated levels of circulating miR-4484 and miR-5196 were detected in the serum of patients with SSc, compared to healthy controls [75,76]. Bioinformatics analysis revealed that miR-4484 could be potentially involved in the TGF-β signaling cascade, ECM-receptor interaction, and MMP expression. Among the latter pathway, the most notable target gene may be MMP-21, which has indeed been observed significantly increased in SSc patients compared to healthy subjects [75]. MiR-5196 exogenous delivery reduced the expression of transcription factor Fra2 (Fos-related antigen-2) and TIMP-1 (tissue inhibitor of metalloproteinases), hinting that it could be useful as a potential regulator of SSc fibrogenesis. In addition, it was also positively correlated with C-reactive protein levels and has been suggested as a biomarker of inflammation in the pathology [75].

Furthermore, other circulating miRNAs resulted upregulated in SSc patients, at plasmatic/serum levels. For example, circulating miR-92a was remarkably elevated in lSSc and dSSc sera and dermal fibroblasts from SSc skin [77]. Authors suggest that miR-92a upregulation may be an effect of TGF-β activation, and the overexpression of this miRNA could induce a downregulation of MMP-1, regulating collagen accumulation [77]. The circulating miR-483-5p was reproducibly observed upregulated in two independent SSc cohorts, including patients with preclinical-SSc symptoms (early SSc) and with localized sclerosis [78]. In particular, its expression correlated with the modified Rodnan skin score (mRSS), in dcSSc subgroup. Its stable expression levels over time, together with its specific upregulation only in SSc disease, and not in other systemic Ads, like SLE and primary SS, suggest its potential role as disease biomarker [78]. Consistently, upregulation of miR-483-5p in fibroblasts and endothelial cells is associated with an increased expression of fibrosis-related genes [65,103].

On the other hand, contrasting results could be observed depending on the type of biofluid investigated, even between serum and plasma samples, as reported for miR-142-3p, a miRNA regulating the expression of integrin αV. Indeed, it was observed significantly higher expressed in serum of both diffuse and limited SSc subjects, compared to healthy controls, and different from other patients’ groups, including SLE, dermatomyositis (DM), or SSD subjects [109]. In contrast, a more recent study reported a decrease of its plasmatic levels in SS patients, compared to controls [110]. In addition to tissue level, miR-21-5p was also found significantly increased at plasmatic level in patients with pulmonary arterial hypertension (APAH), associated with sclerodermic disease [79]. Likewise, upregulated levels of miR-155 were consistently observed also in the sera of SSc patients compared to healthy individuals [80,81]. MiR-155 increase is associated with an early stage of digital microangiopathy, suggesting its potential role as a biomarker for vasculopathy complication on SSc [80].

In order to improve the reliability of circulating miRNAs as potential biomarkers, rather than assessing the abundance and activity of individual molecules, more recent investigations evaluate the expression pattern of multiple miRNAs profile discriminating SSc sera from control individuals, either healthy or with other autoimmune disorders. Following this approach, it has been observed that joint analysis of miR-206 and miR-21 serum levels was more effective to differentiate SSc patients from normal subjects, than single evaluation of miR-206 or miR-21 levels [111]. Another study revealed that miR-223, miR-181b, miR-342-3p, miR-184 were useful in discriminating between diffuse and cutaneous SSc, based on regression analysis and ROC curve evaluation [112]. The analysis of a wider set of miRNAs and study populations evidenced that miRNA-17~92 cluster members (miR-17, miR-20a, miR-92a, and miR-106a) were differentially decreased in SSc sera and can discriminate SSc pathogenic condition from healthy controls. In addition, a set of four opposite regulated miRNAs can be useful in separating SSc (marked by miR-142-3p and miR-223 downmodulation and miR-150 and miR-638 increase) from SLE patients (showing upregulated miR-142-3p and miR-223, together with decrease expression of miR-150, and unaltered miR-638 levels) [110].

MiRNAs-enriched exosomes could be released in the extracellular milieu, having a role in the crosstalk between cells and acting as signaling mediators [95,113]. The number of circulating exosomes was lower in sclerodermic patients, compared to physiological conditions, and a decreased quantity of exosomes has been associated with an increase in vascular complications SSc-related. Considered as a whole, the exosomal fraction isolated from sera of lcSSc and dcSSc patients was found to have an overall profibrotic activity, showing a marked upregulation of 6 profibrotic miRNAs (let-7g, miR-17, miR-23b, miR-155, miR-215, and miR-503), together with decreased levels of 10 antifibrotic miRNAs (let-7a, miR-26b, miR-29b, miR-92a, miR-129, miR-133, miR-140, miR-146a, miR-196a and miR-223) when compared to healthy sera [114]. Interestingly, SSc exosomes induced the expression of profibrotic genes in normal human dermal fibroblasts in vitro treated [114].

## 4. Interplay between Putative Causal Agents of Viral Origin and MiRNAs in SSc

### 4.1. Beta-Herpesvirus Infection and MiRNA Dysregulation in SSc

Several infectious agents have been suggested as potential triggering and/or contributing factors in SSc etiology, in particular those able to persist and occasionally reactivate in the host in adulthood [28,115]. In particular, the most widely studied viruses in association with the disease belong to the human *Orthoherpesviridae* family and *Betaherpesvirinae* subfamily, including HCMV and HHV-6 [41,44,116,117,118]. Both viruses are highly prevalent in the human population, and, commonly to other herpesviruses, after the primary infection in early childhood, they establish a latent infection in the host, with symptomatic reactivation in immunocompromised adults, where they have been associated with a broad range of autoimmune diseases, including connective tissue diseases [117,118,119,120].

Recent observations have confirmed the high prevalence of beta-herpesvirus infection in sclerodermic patients, highlighting HHV-6/HCMV presence both in the tissue and/or blood level (in particular of HHV-6A, which show a prominent tissue tropism), together with significantly enhanced immune response toward viral antigens, such as UL94 (HCMV) and U94 (HHV-6), compared to the healthy population [41,121,122,123,124,125,126]. In order to understand their potential role and their joint involvement in inducing fibrosis, the effects of in vitro infection of primary human dermal fibroblasts (one of the main cell targets affected in SSc pathology, together with endothelial cells) were evaluated [42,43,44,82]. HHV-6A and HCMV in vitro infections induced a rapid and sustained up-modulation of several profibrotic and pro-apoptotic factors, that resulted even more marked by HHV-6A/HCMV coinfection [43,44]. Interestingly, in vitro single and coinfection by those beta-herpesviruses induced a deep modulation of cell miRNome, starting from viral adsorption and early times post infection as well as later times, until 14 days post-infection (d.p.i.). Of note, HCMV and HHV-6A individual infections induced a marked deregulation of a wide set of miRNAs at each tested time, d.p.i., impacting the expression of up to 43 and 29 miRNAs, respectively [42]. The most altered miRNAs upregulated by both viruses and associated with SSc pathways resulted miR-7, miR-let-7g, miR-92a [42].

All these miRNAs were reported upregulated in SSc dermal fibroblasts, and were associated with fibrogenesis, excessive collagen accumulation and pulmonary involvement SSc-related [54,77,108]. In parallel, beta-herpesviruses infection induced a remarkable downregulation of other miRNAs associated with SSc, namely miR-let-7a, miR-10a, and miR-193 [42]. This negative deregulation trend parallels what has been observed in SSc skin biopsies and primary dermal fibroblasts [51,52,72]. Moreover, HCMV infection determined an upregulation of miR-146b, and a downregulation of miR-29, miR-30b and miR-125b. MiR-146b has been previously observed increased in SSc skin tissues and fibroblasts, as well as in fibroblasts and ECs stimulated with SSc patient sera [55]. MiR-29 has a wide reported antifibrotic activity, and it is involved fibrosis repair at different tissue levels, from skin to internal organs such as heart, lung and kidney [127,128,129]. MiR-30b has been suggested as a marker of disease severity, and its amount is inversely correlated with modified Rodnan skin scores and miR-125b [74,107]. The literature data regarding miR-125b highlighted its decreased levels in SSc skin samples and primary dermal fibroblasts, hinting at its potential role as an antifibrotic and antiapoptotic regulator in the disease [54,102]. Last, miR-20 appeared heavily downregulated by HHV-6A infection [42]. Its lower expression in liver fibrosis induced the activation of the TGF-signaling pathway, driving forward extracellular matrix (ECM) production [130]. Interestingly, signaling pathways potentially regulated by HCMV/HHV-6A-induced miRNAs included those related to the fibrotic process, including TGF-β, Wingless/Int (WNT), and β-catenin [42].

HCMV and HHV-6A viruses, highly prevalent in the human population, are likely to infect or reactivate simultaneously in the host. According to this, their synergistic effect on the modulation of miRNAs in coinfected cells has been investigated [82]. Interestingly, the copresence of HCMV and HHV-6A induced a remarkable dysregulation of fibrosis-associated miRNAs expression compared to what was observed in single-infected primary dermal fibroblasts. Overall, a significant upregulation of several profibrotic miRNAs has been observed, coupled in parallel by a likewise pronounced decrease in miRNAs with antifibrotic activity, thereby supporting the profibrotic influence of HCMV/HHV-6A coinfection. Of note, the joint impact on miRNAs modulation involved various miRNAs associated with SSc disease (such as miR-10a, miR-29, and miR-155) but also with other fibrosis-associated diseases, broadening the potential putative implication of HCMV and HHV-6A coinfection in multiple fibrosis-related diseases.

The miRNAs mainly altered in SSc disease, also highlighting the involvement of HCMV and HHV6-A single or double infections, are summarized in Table 1.

### 4.2. Other Virus-Induced MiRNA Dysregulation in SSc

Among the potential viruses associated with the development of SSc, EBV was investigated as a possible trigger. EBV is a human herpesvirus belonging to the *Gammaherpesvirinae* subfamily and was firstly isolated from cultured lymphoblasts derived from a Burkitt’s lymphoma (BL) [132]. Its persistency in latently infected cells has been broadly associated with malignancies, such as endemic BL, Hodgkin’s lymphoma (HL), EBV-positive diffuse large B-cell lymphoma (DLBCL), nasopharyngeal carcinoma (NPC), and EBV-associated gastric carcinoma [133]. In parallel, an increasing body of evidence supports its role in exacerbating and potentially triggering autoimmune and autoinflammatory diseases, including SLE, MS, RA, SS, and even SSc [47,134]. Specifically concerning SSc, EBV infection has been reported since 1981 in some clinical cases [45,46]. Moreover, its infection has been recently detected in monocytes isolated from SSc patients and associated with the activation of Toll-like receptor 8 (TLR8) and the induction of IFN-mediated innate immune response in primary monocytes, suggesting a novel mechanism by which EBV could trigger monocyte inflammation in SSc [47]. Of note, EBV-induced miRNAs were detected and reported to play an important role in maintaining the virus infection and immune evasion [133]. EBV itself encodes for over 40 miRNAs clustered into two genome regions: BART (BamH I-A rightward transcript) and BHRF1 (BamH I-H rightward fragment 1) [135,136,137]. EBV-encoded miRNAs were recognized to support the latent EBV infection, to elude both innate and adaptative immune surveillance of the host, and to promote the neoplastic growth of infected cells [135]. On the other hand, EBV was also reported to induce a deep modulation of multiple host miRNAs, so far mostly recognized as key factors involved in virus-induced oncogenesis and immune escape, including miR-21, the miR-17–92 cluster, and miR-155 [133]. Interestingly, a putative role of EBV-miRNAs in the progression of idiopathic pulmonary fibrosis (IPF) was recently indicated through the induction of epithelial–mesenchymal transition (EMT) in alveolar epithelial cells [138]. Thus, the investigation of EBV-induced miRNAs in a wider range of pathologies, including SSc, could pave the way to a deeper understanding of the mechanisms underlying the autoimmune-based fibrotic processes.

Another virus studied for its potential association with SSc onset is represented by the human Parvovirus B19 (B19V), which is associated with a wide range of pathologies, including erythema infectiosum, arthralgia, fetal death, and autoimmune diseases such as SSc, based on several published studies [139,140,141,142,143,144]. Specifically, SSc patients were observed to show an increased prevalence of B19V viremia and seroprevalence of anti-B19V NS1 antibodies, which are considered a marker of persistent B19V infection [140,141,144]. B19V can infect in vitro dermal fibroblasts and endothelial cells, the main target cells of SSc disease [49,145], together with circulating angiogenic cells (CACs), involved in the vascular regeneration process [146]. At the cellular level, the pathogenic mechanisms by which B19V could cause SSc were linked to the induction of fibrosis (by increased fibroblast activation, migration, and expression of profibrotic factors) [49]; decrease of neovascularization (by the induction of CAC apoptosis) [146], and activation of inflammasome and immune-mediated inflammatory tissue damage [50]. Despite these clues indicating the potential involvement of B19V in the onset or progression of SSc, the role of miRNAs in the virus pathogenesis has been poorly investigated, essentially concerning the regulation of B19V tropism [147,148]; thus, the potential role of B19V-induced miRNAs in SSc has yet to be explored.

### 4.3. Oxidative Stress and MiRNA Dysregulation in SSc

Notably, oxidative stress is often a result of virus infection and has been reported for many virus types, including influenza viruses, hepatitis B virus, human immunodeficiency virus, human coronaviruses, and herpesviruses [149,150,151,152,153,154]. On the other hand, oxidative stress favors herpesvirus infection [155], triggering an imbalance between the increased production of ROS and reduced antioxidant host responses.

The production of ROS and RNS is part of the physiological processes of cellular metabolism, immune response regulation, and inflammation. In several autoimmune diseases, the abnormal production of ROS and RNS, in the proinflammatory status, leads to permanent tissue damages caused by the chemical interactions between reactive compounds and biomolecules such as DNA, proteins, and lipids [40]. Several literature data have highlighted an increased oxidative stress condition in SSc disease [156,157,158,159]. Briefly, higher levels of several oxidative stress biomarkers, including malondialdehyde (MDA), nitric oxide (NO), asymmetric dimethylarginine, and hydroperoxides (ROOH) have been detected in the circulating blood of SSc patients [160]. Increased levels of nitrated proteins and dimethylarginine (ADMA) were observed in dSSc patients and were associated with disease severity and duration [161]. In parallel, the total antioxidant power (TAP) is increased in the serum of SSc patients in response to the increase in oxidative stress [162]. In addition, the enhanced oxidative stress condition has also been detected in SSc urine samples, harboring high urinary levels of 8-isoprostaglandin-F_2α_ (8-iso-PGF_2α_) and hydroxydeoxyguanosine (8-OHdG), biomarkers of free radical damage and DNA oxidative damage, respectively, compared to healthy subjects [163,164].

Lastly, fibrotic SSc skin harbored increased levels of ROS products, together with enhanced nitrotyrosine staining and the excessive abundance of proteins involved in the oxidative stress response, such as peroxiredoxin1 and carbonyl reductase I, compared to control tissues [161,165,166]. Evidence has confirmed the role of oxidative stress in SSc, although the primary cause leading to ROS production is still unclear; some hypotheses support the primary role of NADPH oxidases (NOX)—in particular, NOX2 and NOX4 [167,168].

Other published data have reported a dysregulation of antioxidant systems in SSc disease, affecting, in particular, the levels of CAT, vitamins C and E, and glutathione (GSH), together with the total antioxidant capacity, which was significantly underrepresented in plasma from SSc patients compared to the controls [162,169,170,171].

At the cellular level, pathways involved in oxidative stress and immune responses are finely regulated by miRNAs and vice versa, and ROS/RSN reactive molecules can affect the miRNA levels, showing a close interconnection and mutual regulation among ROS, miRNAs, and inflammation. Several miRNAs have been associated with oxidative stress and immune dysregulation in SSc pathology. To date, the interconnection between miRNA dysregulation and the impairment of the oxidative stress pathway in SSc diseases are still under active investigation.

The main altered miRNAs in SSc involved in the oxidative stress pathways included miR-21, miR-29a, miR-135b, miR-155, miR-182, miR-193b, and miR-200c. Mir-21, together with miR-200c, were found to be increased in SSc patients [58,59,60,89] and mediate the PDCD4 (programmed cell death protein 4)/PTEN (homologous phosphatase and tensin homolog) pathway, resulting in the inhibition of ROS oxidative stress [88,90]. MiR-29a, found decreased in SSc [58,59,71], and target SIRT1 (silent information regulator factor 2–related enzyme 1) histone deacetylase can deacetylate the FoxO factors and consequently regulate the expression of antioxidant enzymes such as superoxide dismutase (SOD), catalase (CAT), and thioredoxin (TRX) [91]. In addition, miR-29 is also reported to target insulin grow factor 1 (IGF1), which is an upstream activator of the AKT (protein kinase B) signaling pathway, through phosphatidylinositol 4,5-bisphosphate (PIP2) conversion [172]. BMP-7 (bone morphogenetic protein 7), another upstream regulator of the AKT pathways, is instead regulated by miR-135b [92]. Since miR-135b also negatively regulates USP15, a deubiquitinase involved in the Keap1/Nrf2 antioxidative pathway [83], its decreased levels in SSc may also affect the protective effect of this pathway on oxidative stress. MiR-155 has a well-known role as inflamma-miR, finely regulating the NF-κB pathway [173], and its upregulation has been reported in sclerotic lesions of different tissues [66,67,131]. In addition, the knockdown of miR-155 could regulate ROS production, NO generation, and apoptosis via different pathways, including those mediated by PI3K/Akt [174]. As already mentioned, miR-193b contributes to proliferative vasculopathy in SSc, mediated by uPA regulation [51]. Lastly, another miRNA, miR-182, has been described as involved in oxidative stress regulation, being upregulated in SSc patients [85]. It could have a bivalent role, both inhibiting NOX4 and thus decreasing ROS production or upregulating the intracellular redox status via downmodulation of the antioxidant modulator sestrin2 (SESN2) [86,87].

## 5. Future Directions

The rapid development of omics analysis technologies, including next-generation sequencing and other high-throughput methods, allows the genome-wide investigation of miRNome defining healthy and disease conditions, including Systemic Sclerosis. The characterization of dysregulated miRNA expression levels, which either define SSc pathology or are induced by environmental stimuli, such as oxidative stress and viral infection, provides insight into the molecular mechanisms primarily involved in disease onset, progression, and exacerbation. Among the most advanced analytical methods applied to detect and quantify the miRNAs levels, miRNA sequencing has assumed a growing interest, allowing also the identification of novel miRNAs and pre-miRNAs, unlike the qRT-PCR and miRNA array methods [175]. To date, public databases have more than two thousand miRNAs registered, highlighting the potential of a bioinformatics-integrative approach for genome-wide identification and miRNA–mRNA gene prediction [176,177,178].

A further improvement of the miRNome investigation involves the study of a single cell rather than a bulk of cells constituting tissue biopsies and PBMC fractions. Epigenetic regulation often involves specific cell subpopulations of immune cells or cell types within the same tissue (i.e., ECs vs. myofibroblasts in skin biopsy). Single-cell technologies, such as mass cytometry, cell sorting (FACS), single-cell RNA sequencing, and spatially resolved transcriptomics, represent cutting-edge technologies that enable the comprehensive characterization of dynamic epigenetic changes with single-cell resolution [179,180].

As a result of these new approaches, the increasing knowledge of the miRNA-regulated molecular mechanisms has opened the way to the design of innovative therapeutic approaches. MiRNAs could be investigated as therapeutics (miRNA mimics) or as targets of therapeutics (anti-miRNAs) in order to inhibit the transcription of multiple target genes [181]. Although a promising use of miRNA-based therapeutic tools, the main challenges to overcome include the optimization of proper administration routes, the control of delivery in the blood, the specific targeting of tissues and cell types, and the minimization of adverse effects. Given these multiple aspects still to be optimized, only a few miRNA-based drugs have been evaluated in a clinical test phase so far [181].

On the other hand, circulating miRNAs are stable, less susceptible to degradation than RNA, and easily detectable and quantifiable by sensitive methods in different types of body fluids, first and foremost being blood and plasma [23,79,94,114]. These characteristics make them reliable biomarkers for the diagnosis and discrimination of several autoimmune diseases, including SSc, as demonstrated in several studies so far [79,107,109,110,112,182,183]. However, their use as biomarkers is associated with downsides that need to be addressed—in particular, the choice of biological fluid and the appropriate method of collection, the potential contamination of intracellular RNA, the choice of the most sensitive and potentially standardizable analytical method, and last, the method of data normalization [184,185]. In conclusion, rigorous trials on a large study population will be needed to validate the diagnostic potential of these molecules. Based on the available data so far, general conclusions are still difficult to draw, since a solid interpretation is still hampered by the limitations described for each individual study. Thus, despite the large amount of collected data, future research would be needed to confirm the data in studies conducted in larger cohorts and by using standardized procedures in order to achieve sound and reliable results specifically targeted to SSc and useful for clinical applications.

## 6. Conclusions

Systemic Sclerosis is a multifaceted autoimmune disease characterized by vascular complications, immune dysregulation, and an excessive fibrosis affecting multiple organs. Although, in recent years, an earlier diagnosis and the routine examination of organ involvement have improved treatment and overall survival, the therapeutic options for SSc are mainly symptom- and organ-based. The little knowledge of the causative factors and the high complexity of the disease result in the lack of an effective treatment for SSc. The research on miRNA modulation in SSc patients, together with that induced in infected cells by the putative SSc causal factors such as beta-herpesviruses, has opened a new perspective on our options to understand the mechanisms by which specific agents could contribute to the onset and progression of the disease. Based on available data, some miRNAs emerged as potential profibrotic factors, both detectable in SSc patients and in beta-herpesvirus infected cells (i.e., miR-let-7, miR-146, and miR-155). From this perspective, miRNAs could serve as novel biomarkers for the rapid end sensitive SSc diagnosis and could represent promising therapeutic targets for SSc treatment.

## Figures and Tables

**Table 1:** MiRNAs deregulated in SSc disease and potential beta-herpesvirus involvement: expression levels, localization, and functional role.

miRNA	Tissue/Cell Type	Effect	Expression (SSc vs. CTR)	Refs. No.
miR-7 *	LSc dermal fibroblasts, serum	Induces overexpression of α2(I) collagen	up/down	[72]
miR-10a *	Skin biopsy, fibroblasts	Decreases in EC cells stimulated with SSc serum	down	[55]
miR-20 *	Dermal fibroblasts	Activated TGF-β pathway toward ECM production	down	[130]
miR-21	Skin biopsy, lung and dermal fibroblasts, mouse model, plasma	Enhances TGF-β signaling events in SSc fibrosis targeting SMAD7 Role in immune activation	up	[58,79,100,101]
miR-26b	Fibroblast	Promotes cell fibrosis markers expression targeting CXCL9 and CXCL13	up	[68]
miR-27a	Lung and skin biopsy	Promotes fibrotic gene expression by SPP1/ERK1/2 axis	down	[56]
miR-29a	Serum	Dysregulates type I collagen deposition, targeting COL1A1 and COL1A	down	[71]
miR-29b *	Skin biopsy, fibroblasts	Regulates TGFβ pathway targeting COL1A1	down	[58]
miR-30b *	Skin biopsy, mouse model, serum	Promotes fibrosis by regulating PDGFR-β	down	[74]
miR-92a *	Dermal fibroblasts, serum	Decreases MMP-1 expression and control excessive collagen accumulation	up	[77]
miR-125b *	Skin biopsy, fibroblasts	Induces apoptosis, hindered proliferation and α-SMA expression	down	[102]
miR-126	pDCs	Regulates pDC activation via TLR9-mediated response and IFN signaling	up	[69]
miR-130	Skin biopsy, fibroblasts	Promotes TGF-β pathway via antifibrotic PPARγ inhibition	up	[62]
miR-135b	Fibroblast, serum	Regulates USP15, involved in Keap1/Nrf2 antioxidative pathway	down	[83]
miR-139	pDCs	Regulates pDC activation via TLR9-mediated response and IFN signaling	up	[69]
miR-142	Serum, plasma	Regulates the expression of integrin αV	up in serum/down in plasma	[109,110]
miR-145	Skin biopsy, fibroblasts	Regulates TGF-β pathway targeting SMAD3	down	[58]
miR-146b *	Skin biopsy, fibroblasts	Increases in EC cells stimulated with SSc serum	up	[55]
miR-150	Skin biopsy, fibroblasts, serum	Induces overexpression of type I collagen and integrin β3 by TGF-β activation	down	[57]
miR-155 *	Skin biopsy, lung fibroblast, mouse model, serum	Regulates of Wnt/β- catenin and Akt pathways Enhances inflammation, apoptosis, and oxidative stress Potential biomarker for vasculopathy	up	[66,67,80,81,84,131]
miR-182	Dermal fibroblast	Regulates oxidative stress with bivalent effect	up	[85,86,87]
miR-193b *	Skin biopsy, fibroblasts	Regulates proliferative vasculopathy by uPA overexpression	down	[51]
miR-196a	LSc skin biopsy, serum	Induces overexpression of type I collagen	down	[73]
miR-200c	PBMC	Inhibits ROS oxidative stress via PDCD4/PTEN	up	[88]
miR-202	Skin biopsy, fibroblasts	Promotes fibrosis by regulation of MMP1 expression	up	[61]
miR-206	Serum	Could discriminate SSc from ctr sera, together with miR-21	down	[111]
miR-320a	Pulmonary fibroblasts, mouse model	Inhibits collagen deposition targeting TGFBR2 and IGF1R	down	[63]
miR-483	Fibroblasts, endothelial cells, serum	Promotes collagen IV-encoding genes COL4A1- COL4A2, and transition of ECs to a myofibroblast phenotype	up	[65,78]
miR-618	pDCs	Inhibits pDCs development and induces IFNα secretion	up	[70]
miR-3606	Skin biopsy, fibroblasts	Hinders TGF-β pathway targeting TGFβ-receptor TGFBR2	down	[64]
miR-4484	Serum	Involved in TGF-β signaling pathway, ECM-receptor interaction, and MMP expression	up	[75]
miR-5196	Serum	Regulates fibrogenesis targeting FRA2 gene	up	[76]
let-7 family	Skin biopsy	Involved in pulmonary hypertension in SSc	down	[53]
let-7a *	Skin biopsy, serum	Induces excessive expression of type I collagen	down	[52]
let-7g *	Skin biopsy	Correlates with SSc pathogenesis	up	[54]
miR-223 miR-342 miR-181b miR-184	Serum	This miRNAs cluster could discriminate dSSc from lSSc sera	down (dSSc vs. lSSc) up dSSc vs. lSSc)	[112]
miR-17 miR-20a miR-92a miR-106a	Serum	This miRNAs cluster could discriminate SSc from ctr sera	down	[110]
miR-142 miR-223 miR-150 miR-638	Serum	This miRNAs cluster could discriminate SSc from SLE sera	down (SSc vs. SLE) up (SSc vs. SLE)	[110]
let-7g miR-17 miR-23b miR-155 miR-215 miR-503	Serum exosomes	profibrotic miRNAs SSc exosomes induce a profibrotic phenotype in cultured dermal fibroblasts	up	[114]
let-7a miR-26b miR-29b miR-92a miR-129 miR-133 miR-140 miR-146a miR-196a miR-223	Serum exosomes	antifibrotic miRNAs SSc exosomes induce a profibrotic phenotype in cultured dermal fibroblasts	down	[114]

* Asterisks indicate miRNAs affected by HHV-6A and/or HCMV infection [42,82] (see Figure 1).
